# Factors associated with COVID-19 vaccine acceptance among medical laboratory workers in South Africa

**DOI:** 10.4102/jphia.v16i1.1291

**Published:** 2025-10-28

**Authors:** Melitah Motlhale, Kerry Wilson, David Jones, Graham Chin, Nisha Naicker

**Affiliations:** 1 Epidemiology and Surveillance Section, National Institute for Occupational Health, National Health Laboratory Service, Johannesburg, South Africa; 2 School of Public Health, Faculty of Health Sciences, University of the Witwatersrand, Johannesburg, South Africa; 3 Department of Safety Health and Environment (SHE), National Institute for Occupational Health, National Health Laboratory Service, Johannesburg, South Africa; 4 Department of Environmental Health, Faculty of Health Sciences, University of Johannesburg, Johannesburg, South Africa

**Keywords:** COVID-19 vaccine, vaccination hesitancy, vaccination acceptance, vaccines, South Africa

## Abstract

**Background:**

During the COVID-19 pandemic, medical laboratory workers had a higher risk of contracting COVID-19 compared to the general population.

**Aim:**

To assess the COVID-19 vaccine acceptance and hesitancy among medical laboratory workers in South Africa in 2022.

**Setting:**

In South Africa among the healthcare worker population at the National Health Laboratory Service (NHLS) in 2022.

**Methods:**

Descriptive statistics was used to identify the reasons for COVID-19 acceptance. We examined the association between COVID-19 acceptance and other socio-demographic factors using logistic regression analyses to calculate the odds ratio (OR) and 95% confidence interval (CI).

**Results:**

The prevalence of COVID-19 vaccine acceptance among NHLS workers was 82.8%. Most of the participants reported that their reason for COVID-19 vaccine acceptance was mainly to protect their family (62.6%) and to protect themselves (50.2%), and they perceived the vaccine to be safe (40.7%). COVID-19 vaccine hesitancy was mainly because the participants reported that there was very little research done on the vaccine (41.4%) and some were worried about the vaccine side effects (31.4%). Increased COVID-19 vaccine acceptance was associated with age, 40–49 years (OR: 5.35 [95% CI: 1.42–20.10]) and 50–59 years (OR: 11.0 [95% CI: 1.63–74.92]). Decreased COVID-19 vaccine acceptance was associated with black people (OR: 0.15 [95% CI: 0.03–0.89]).

**Conclusion:**

The prevalence of COVID-19 vaccine acceptance among medical laboratory workers was notably high.

**Contribution:**

This study contributes to the body of knowledge on vaccine acceptance and hesitancy.

## Introduction

The COVID-19 is an infectious disease caused by the severe acute respiratory syndrome coronavirus 2 (SARS-CoV-2). COVID-19 was officially declared a pandemic by the World Health Organization (WHO) on 11 March 2020 and unfolded with over 153 million confirmed cases and approximately 2 million deaths globally by January 2021.^1^ The pivotal role of medical laboratory workers on the pandemic’s frontlines became evident, as they faced an escalated risk of contracting the disease.^2^ A study in the United Kingdom (UK) and the United States (US) revealed that medical laboratory workers were nearly 12 times more likely to acquire COVID-19 compared to the general population.^3^ In South Africa, medical laboratory workers made up almost 3% of COVID-19 hospital admissions as of April 2021.^4^

Amid the absence of effective vaccines and treatments, global efforts focused on implementing preventative measures, including handwashing, social distancing and mask-wearing.^5^ As of February 2021, various COVID-19 vaccines had received emergency use approval worldwide. South Africa (SA) prioritised healthcare workers (HCWs) for vaccination because of their elevated risk and essential role in patient care.^6,7^ The initial phase of the country’s vaccine rollout programme commenced among frontline HCWs on 17 February 2021.^7^

The introduction of effective vaccines played a crucial role in reducing the virus’s spread and mitigating the COVID-19 impact.^8^ However, the success of vaccination programmes globally faced challenges related to vaccine hesitancy, influenced by factors such as religion, culture, politics, media and previous vaccination experiences.^9^ In SA, a study reported a high prevalence of vaccine hesitancy (41%) among a diverse group of HCWs.^10^ This mirrored the findings from a US systematic review reporting a 40.8% vaccine hesitancy prevalence among HCWs.^11^ Interestingly, some African countries, including Zambia, showed notably high acceptance (72.1%) of the COVID-19 vaccine among HCWs.^12^ In 2019, the WHO identified vaccine hesitancy as a significant global health threat.^13^ The conceptual framework on drivers of vaccine hesitancy encapsulates the concepts suggested by literature.^14^ The conceptual framework identified four domains being individual perceptions, social influences, COVID-19 vaccine and related factors and COVID-19 infection and related factors. These domains contribute to vaccine acceptance and hesitancy.

The reasons and factors associated with vaccine acceptance and hesitancy among medical laboratory workers in South Africa are not well studied. Understanding vaccine acceptance and hesitancy will highlight the gaps in vaccination campaigns and contribute to the development of targeted training programmes and interventions. Our study aimed to identify reasons for COVID-19 vaccine acceptance and hesitancy and to identify factors associated with COVID-19 vaccine acceptance among South African medical laboratory workers, recognising their heightened risk for COVID-19 infection compared to the general population.

## Research methods and design

### Study design and data sources

This was an analytical cross-sectional study using secondary data from the COVID-19 surveillance in a HCW population study conducted at the National Health Laboratory Service (NHLS) in 2022. We extracted data on the primary study’s demographic characteristics and reasons for vaccine acceptance and hesitancy among medical laboratory workers.

### Study population

This study included medical laboratory workers of working age (20–65 years). Staff members were categorised into four occupational groups: administrative and clerical, laboratory staff, medical staff and other staff members. Administrative and clerical staff included clerk (laboratory-based) laboratory assistant, administrators including human resources and finance or clerk (non-laboratory) and management staff (non-laboratory), laboratory staff (medical technician, medical technologist, medical scientist, laboratory manager, intern or student and information technology), medical staff (phlebotomist, medical specialist, pathologist, registrar and nurse) and other (driver or messengers, general workers including cleaner, security staff and workshop and others).

### Data collection

All medical laboratory workers were eligible for the study. For this study, no sampling was done, every employee received an email, except those without email addresses. We used a voluntary online self-administered questionnaire to collect data. Participants were invited to participate in the study via their work email address with a REDCap link. All NHLS employees received two email reminders to complete the questionnaire because of poor response. Those who responded were stratified by occupational groups, and a research assistant called randomly selected individuals from each of the main occupational groups. A random selection of staff received telephone calls to remind them to participate. A research assistant conducted telephonic interviews with those staff without access to work email address, such as cleaners, messengers and general workers. The questionnaire collected participants’ demographic information, including work region, chronic medical conditions and previous vaccinations. Information was collected on COVID-19 infections and vaccination along with reasons for acceptance or hesitancy. We managed to collect data from 422 medical laboratory workers in August 2022 (after the vaccine rollout).

### Data management

The statistical analysis included age, categorised into a 10-year age band (< 30 years, 30–39 years, 40–49 years, 50–59 years, 60+ years) and sex coded as male or female. Race was self-reported as Asian, black African, coloured, Indian, white and other; for analysis purposes, we collapsed the categories into black people, white people and others, because of small numbers. Work location area was categorised as rural or urban, and the occupational groups were included; administrative and clerical (clerk [laboratory based], laboratory assistant, admin including human resources and finance or clerk and management staff [non-laboratory]), laboratory staff (medical technician, medical technologist, laboratory manager, intern or student, information and technology and medical scientist), medical staff (phlebotomist, medical specialist, pathologist, registrar and nurse) and other (driver or messenger, general worker including cleaners and other jobs).

COVID-19 history included individuals who reported testing positive for COVID-19 at any point before or during 2022. National Health Laboratory Service intranet training was both general information on COVID-19 and COVID-19 vaccination. Participation in the Occupational Health and Safety Information System (OHASIS) COVID-19 symptom screening was a workplace legislated requirement where employees who had symptoms of COVID-19 needed to report them to the employer. Prior vaccination was defined to include participants who received vaccines other than the COVID-19 vaccine, including varicella, influenza, hepatitis B, BCG (Bacillus Calmette-Guérin), MMR (measles-mumps-rubella) and TDap (tetanus, diphtheria, pertussis). Participants also self-reported on whether they experienced stress at home or work.

Vaccine hesitancy is defined as the delay in acceptance, reluctance or refusal of vaccination despite the availability of vaccination services.^15^ In this study, individuals who received the COVID-19 vaccine were categorised as ‘accepting’, whereas those who did not receive the vaccine but who were eligible were classified as ‘hesitant’ towards the vaccine.

### Statistical analyses

All the data collected during the cross-sectional survey were exported to Stata (2019) version 16 (StataCorp, US) for analysis. Descriptive statistics were used to identify the reasons for COVID-19 vaccine acceptance and hesitancy and describe frequency. To ascertain the factors associated with vaccine hesitancy among medical laboratory workers in SA, a Chi-square test was performed to investigate the possible relationship between demographic characteristics and vaccine hesitancy among the participants. A univariate logistic regression analysis followed by a multivariate analysis was also employed to identify the relationships between the independent variables (sex, age, job category, chronic medical conditions, COVID-19 history and stress) and the dependent variable (COVID-19 vaccination status). A *p*-value of less than 0.05 was considered statistically significant. Results are presented as odds ratios (OR) with their 95% confidence intervals (CI).

### Ethical considerations

The University of the Witwatersrand Human Research Ethics Committee (Medical) approved the primary study and the current study (Clearance certificate number: M211138). The approval to conduct the study at the NHLS was obtained from the Academic Affairs Research and Quality Assurance office (Reference number: PR2119473). Written informed consent was obtained from all participants.

## Results

A total of 422 participants participated in the study. At the time of the study, the company had approximately 8400 staff members, giving this survey a response rate of 5%. Of the 422 participants, 407 (96.4%) answered the question ‘Have you been vaccinated for COVID-19?’ Approximately 82.8% of medical laboratory workers reported that they had been vaccinated for COVID-19, while 17.2% indicated that they were not vaccinated for COVID-19 (Table 1). The majority of the participants were females (70.7%) similar to the 68% of women employed at the company in 2022. The most common population group was black African people (56.8%), those aged between 30 years and 39 years (35.5%), those who worked in facilities based in urban areas (77.6%) and 53.2% were laboratory staff. A significant difference in acceptance of the vaccine was found in three categories of age group (*p* = 0.001) and race (*p* = 0.040) (Table 2).

**TABLE 1:** Demographic characteristics and COVID-19 vaccine acceptance and hesitancy among the participants (*N* = 407).

Variable	Vaccine acceptance†				Total		*p*-value
	No		Yes		*n*	%	
	*n*	%	*n*	%			
**Sex**	-	-	-	-	-	-	0.474*
Female	47	67.1	240	71.4	287	70.7	-
Male	23	32.9	96	28.6	119	29.3	-
**Age group (years)**	-	-	-	-	-	-	-
< 30	9	12.9	22	6.6	31	7.6	**0.0729**
30–39	37	52.9	107	31.9	144	35.5	**0.0008**
40–49	13	18.6	106	31.6	119	29.3	**0.0309**
50–59	7	10.0	76	22.6	83	20.4	**0.0174**
60+	4	5.7	25	7.4	29	7.1	0.6091
**Race**	-	-	-	-	-	-	-
Black people	51	73.9	179	53.3	230	56.8	**0.0015**
White people	10	14.5	90	26.8	100	24.7	**0.0299**
Other	8	11.6	67	19.9	75	18.5	0.1035
**Facility location**	-	-	-	-	-	-	0.467*
Rural	18	25.7	73	21.7	91	22.4	-
Urban	52	74.3	263	78.3	315	77.6	-
**Occupational group**	-	-	-	-	-	-	0.258*
Administrative and clerical staff	20	28.6	68	20.2	88	21.7	-
Laboratory staff	38	58.3	178	53.0	216	53.2	-
Medical staff	7	10.0	54	16.1	61	15.0	-
Other	5	7.1	36	10.7	41	10.1	-

Note: Bold values are the statistically significant *p*-value.

* , Chi-square test *p*-value.

† , No: *n* = 70 (17.2%); Yes: *n* = 337 (82.8%).

**TABLE 2:** COVID-19-related factors and other factors influencing vaccine acceptance and hesitancy among the participants (*N* = 407).

Variable	Vaccine acceptance†				Total		*p*-value
	No		Yes		*n*	%	
	*n*	%	*n*	%			
**COVID-19 histor y**	-	-	-	-	-	-	-
No	34	49.3	135	40.7	169	42.1	0.357
Yes	35	50.7	195	58.7	230	57.4	0.134
Not sure	0	0.0	2	0.6	2	0.5	-
**NHLS intranet training**	-	-	-	-	-	-	**0.016**
No	31	44.9	100	29.9	131	32.5	-
Yes	38	55.1	234	70.1	272	67.5	-
**OHASIS COVID-19 symptom screening**	-	-	-	-	-	-	0.951
No	25	35.7	118	35.3	143	35.4	-
Yes	45	64.3	216	64.7	261	64.6	-
**All doses (completed the vaccine schedule)**	-	-	-	-	-	-	**< 0.001**
No	-	-	68	20.2	68	20.2	-
Yes	-	-	269	79.8	269	79.8	-
**Booster vaccine**	-	-	-	-	-	-	**0.0108**
No	-	-	134	43.2	134	43.2	-
Yes	-	-	176	57.8	176	57.8	-
**Chronic medical condition**	-	-	-	-	-	-	0.188*
No	48	69.6	211	63.6	259	64.6	-
Yes	16	23.2	108	32.5	124	30.9	-
Don’t know	5	7.3	13	3.9	18	4.5	0.227*
**Prior vaccination**	-	-	-	-	-	-	-
No	5	7.1	41	12.2	46	11.3	-
Yes	65	92.9	296	87.8	361	88.7	-
**Stress at home**	-	-	-	-	-	-	0.266*
No	63	90.0	281	84.9	344	85.8	-
Yes	7	10.0	50	15.1	57	14.2	-
**Stress at work**	-	-	-	-	-	-	0.065*
No	44	62.9	168	50.8	212	52.9	-
Yes	26	37.1	163	49.2	189	47.1	-

Note: Bold values are the statistically significant *p*-value.

NHLS, National Health Laboratory Service; OHASIS, Occupational Health and Safety Information System.

* , Chi-square test *p*-value.

† , No: *n* = 70 (17.2%); Yes: *n* = 337 (82.8%).

Most participants reported a history of COVID-19 (57.4%), they had engaged with NHLS intranet training (67.5%) and participated in the OHASIS COVID-19 symptom screening (64.5%). Participants reported no chronic medical conditions (64.6%), and 88.7% had a prior vaccination history. There was no significant difference in the acceptance of the COVID-19 vaccine by sex, job category, COVID-19 history, chronic medical condition, prior vaccinations, stress at home and stress at work (> 0.05). Of those who were vaccinated for COVID-19, 79.8% completed the vaccine schedule and 57.8% reported taking the booster vaccine.

### Reasons for COVID-19 vaccine acceptance versus reasons for hesitancy among medical laboratory workers

**FIGURE 1:**
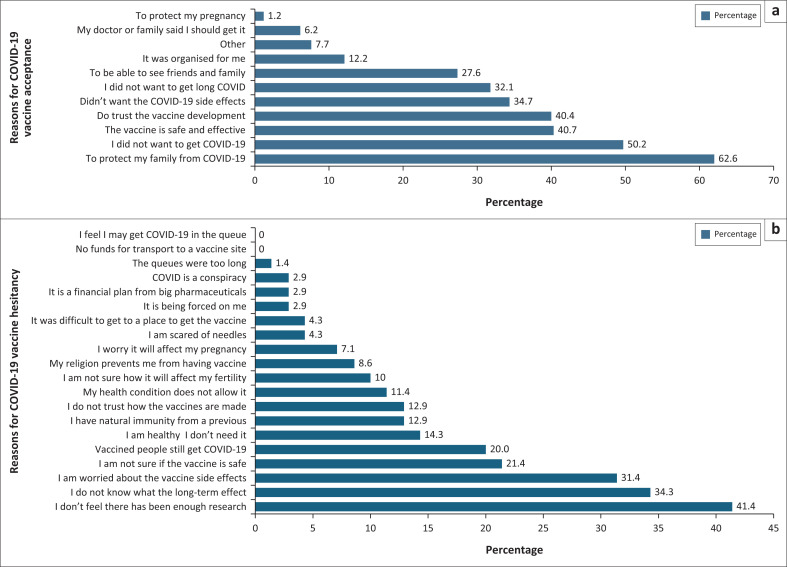
(a and b) Reasons for COVID-19 vaccine acceptance and hesitancy among the study participants.

About 62.6% (*n* = 211) of the participants reported that they vaccinated for COVID-19 to protect their families from COVID-19, 50.2% to prevent contracting COVID-19 infection, 40.7% of the participants believed that the vaccine was safe and effective, and 40.4% trusted the research done in developing the vaccine (Figure 1a). The least number of participants reported on taking the vaccine to protect their pregnancies (1.2%).

Among participants who were not vaccinated, the top reasons at 41.4% for hesitancy included feeling like there had not been enough research done on the COVID-19 vaccine, 34.3% reported being unsure about the long-term effects of the vaccine and 31.4% were concerned about the side effects caused by the vaccine. No participants indicated that not having enough funds for transport to get to the vaccination site or feeling there was a risk of getting infected in the queues was the reason for not vaccinating (Figure 1b).

### Factors associated with COVID-19 vaccine acceptance among medical laboratory workers

The bivariate logistic regression results showed that medical laboratory workers between the ages 40–49 years (OR: 3.34 [95% CI: 1.27–8.76]) and 50–59 years (OR: 4.44 [95% CI: 1.48–13.29]) were more likely to be vaccinated for COVID-19 compared to participants less than 30 years of age. Black people (OR: 0.39 [95% CI: 0.19–0.80]) were less likely to be vaccinated compared to white people. The multivariate analysis did not highlight age and race as predictors for vaccine acceptance among medical laboratory workers (Table 3). Increased COVID-19 vaccine acceptance was associated with age: 40- to 49-year-olds (OR: 5.35 [95% CI: 1.42–20.10]), 50- to 59-year-olds (OR: 11.0 [95% CI: 1.63–74.92]). Decreased COVID-19 vaccine acceptance was associated with being black people (OR: 0.15 [95% CI: 0.03–0.89]).

**TABLE 3:** Factors associated with COVID-19 vaccine acceptance versus hesitancy among medical laboratory workers.

Variable	Univariate			Multivariate		
	OR	95% CI	*p*-value	OR	95% CI	*p*-value
**Sex**						
Female	1.00	-	-	-	-	-
Male	0.82	0.47–1.42	0.474	-	-	-
**Age group (years)**						
< 30	1.00	-	-	1.00	-	-
30–39	1.18	0.50–2.80	0.702	1.32	0.50–3.48	0.579
40–49	**3.34**	**1.27–8.76**	**0.015**	**5.35**	**1.42–20.10**	**0.013**
50–59	**4.44**	**1.48–13.29**	**0.008**	**11.0**	**1.63–74.92**	**0.014**
60+	2.56	0.69–9.47	0.160	8.66	0.69–108.34	0.094
**Race**						
Black people	**0.39**	**0.19–0.80**	**0.011**	**0.15**	**0.03–0.89**	**0.036**
White people	1.00	-	-	1.00	-	-
Other	0.93	0.35–2.48	0.886	5.33	0.72–39.75	0.103
**Facility location**						
Rural	1.00	-	-	-	-	-
Urban	1.25	0.69–2.26	0.467	-	-	-
**Occupational group**						
Administrative and clerical staff	1.00	-	-	-	-	-
Laboratory staff	1.38	0.75–2.53	0.303	-	-	-
Medical staff	2.27	0.89–5.76	0.085	-	-	-
Other	2.12	0.73–6.11	0.165	-	-	-
**Chronic medical condition**						
No	1.00	-	-	1.00	-	-
Yes	1.54	0.83–2.83	0.169	0.99	0.50–1.94	0.968
**COVID-19 history**						
No	1.00	-	-	1.00	-	-
Yes	1.40	0.83–2.36	0.202	1.60	0.89–2.86	0.118
**Prior vaccination**						
No	1.00	-	-	-	-	-
Yes	0.56	0.21–1.46	0.233	-	-	-
**Home stress**						
No	1.00	-	-	-	-	-
Yes	1.60	0.69–3.70	0.270	-	-	-
**Work stress**						
No	1.00	-	-	1.00	-	-
Yes	1.64	0.97–2.79	0.067	1.00	0.83–2.71	0.177

Note: Data in bold are statistically significant OR (95% CI). Statistically significant *p*-value < 0.05.

OR, odds ratio; CI, confidence interval.

## Discussion

Numerous studies globally have investigated HCWs’ attitudes towards COVID-19 vaccines, yielding conflicting results.^10,12,16,17,18,19^ Similar disparities have been observed in studies that assessed the attitudes of HCWs towards the COVID-19 vaccine in South Africa.^10,16,19^ Given HCWs’ pivotal role in combating COVID-19, addressing vaccine hesitancy among them is imperative.

The present cross-sectional study demonstrates high COVID-19 vaccine acceptance among medical laboratory workers, with nearly 80% having been vaccinated at the time of the study, aligning with findings from other studies conducted among South African HCWs, which reported a 90% and 89% acceptance rate, respectively.^16,19^ Conversely, vaccine hesitancy prevalence of 17% resembled that of the general population in South Africa (24%) as reported by a South African National Dynamics Study in 2021. However, it was considerably lower than that reported among HCWs in Cape Town (41%).^10,20^ Variability in acceptance rates, as reported in many South African studies and corroborated by a review by Cooper et al., underscores the evolving public perception influenced by available information and other factors.^21^

Across diverse contexts, multiple factors influence both vaccine acceptance and hesitancy, with age playing a notable role. Our findings are similar to a recent study in Kenya, showing that the drivers of vaccine acceptance and hesitancy are complex.^14^ Numerous studies have highlighted age as a crucial predictor of vaccine hesitancy and acceptance.^10,14,18,22,23^ Several research results indicate that younger age groups show higher levels of vaccine hesitancy, aligning with our findings where older HCWs were more inclined to receive the COVID-19 vaccine compared to their younger counterparts.^10,22,23^ This trend may stem from younger individuals being disproportionately influenced by misinformation circulated about the vaccine across diverse social media platforms.^24^ Notably, previous research underscored the prevalence of misleading vaccine information on platforms such as YouTube and Twitter that young people who are active on these platforms are exposed to.^25,26^ Furthermore, it has been shown that younger individuals often perceive a lower risk of severe COVID-19 infection compared to older age groups.^14,27^

Even though stress at home or at work was not a predictor for COVID-19 vaccination in our study, a study in Japan reported that stress, particularly work-related stress, served as a motivating factor for HCWs to seek influenza vaccine uptake and COVID-19 vaccination intention.^28^ This association is believed to be linked to factors such as inadequate personal protective equipment (PPE) and perceptions of workplace infection control policies, which contribute to heightened work stress and consequently a stronger inclination towards COVID-19 vaccination.^28^

While the participants’ profession was not a significant predictor of vaccine acceptance in our study, it has been a notable factor in other research contexts.^10,17,29^ A British review highlighted that nurse and non-clinical HCWs showed lower acceptance rates of the COVID-19 vaccine, citing concerns regarding safety, efficacy, effectiveness and governmental trust as major barriers.^29^ Similarly, other studies found that HCWs, including laboratory technologists, radiography technologists and pharmacy professionals, displayed more hesitancy towards COVID-19 vaccination compared to nurses and doctors.^17,27,30^ This indicates that individuals with lower direct patient contact perceived a reduced risk of COVID-19 exposure. However, it is crucial to acknowledge that the majority of COVID-19 infections among HCWs were attributed to occupational exposures in non-COVID-19 facilities, as highlighted in studies by Alajmi et al.^31^ Inadvertent exposure to infected colleagues within various departments accounts for nearly half of the infections in the workspace, which can be more than those originating from patients.^32^ Additionally, a study in Oman suggested that a significant proportion (61%) of HCW infections were acquired within the community.^33^ These findings underscore the importance of comprehensive infection control measures both within healthcare settings and the broader community to mitigate the risk of COVID-19 transmission among HCWs.

Previous vaccination has also been associated with vaccine acceptance,^23,24,34^ and our results contradict these findings. Research suggests that individuals who have received prior vaccines, such as the flu vaccine, are more likely to accept the COVID-19 vaccine, possibly because of established trust in vaccine safety.^23,24,35^ In our study, many hesitant participants expressed distrust in the safety of the COVID-19 vaccine, citing concerns about inadequate research and apprehensions regarding potential side effects and long-term impacts. Conversely, most participants who had received the COVID-19 vaccine expressed confidence in the research in developing the vaccine. These findings align with other studies highlighting the significance of trust in vaccine safety, efficacy and governmental or health authority recommendations in influencing COVID-19 vaccine hesitancy.^23,34,36^

Most regulatory trials for COVID-19 vaccines excluded individuals with chronic medical conditions although some studies included a limited number of patients with cancer and autoimmune diseases.^37^ Consequently, the availability of limited vaccine safety data may lead to conflicting attitudes towards vaccination among vulnerable populations.^37^ Our study found that while the presence of a chronic medical condition was not a significant predictor of vaccine acceptance, participants with such conditions were nearly twice as likely to receive the COVID-19 vaccine compared to those without any chronic medical condition. However, a survey of individuals with serious comorbid conditions revealed that one in five respondents reported COVID-19 vaccine hesitancy.^37^ The exclusion of immunocompromised individuals from regulatory clinical trials may have contributed to safety concerns expressed by 44% of vaccine-hesitant respondents.^38,39^ Therefore, assumptions that the most vulnerable would automatically accept COVID-19 vaccination are unfounded, highlighting the need for healthcare team members to initiate discussions focusing on the vaccine’s impact on an individual’s underlying condition.

The majority of medical laboratory workers who received the COVID-19 vaccine expressed a desire to safeguard themselves and their families against COVID-19 infection. Therefore, it is imperative to educate the public about the vaccine’s effectiveness and the concept of herd immunity, as these are pivotal factors in promoting vaccination. Additionally, medical laboratory workers require comprehensive education on the extensive research conducted on the COVID-19 vaccine, as many non-vaccinated individuals expressed concerns about the adequacy of safety research.

Accessible and reliable sources of vaccination information must be provided to HCWs, as misinformation circulating on social media platforms can influence their perceptions of vaccine safety. A study conducted among HCWs in Dubai revealed that only 38% of primary care HCWs relied on scientific journals or research papers as their primary sources of information during the pandemic.^40^ Furthermore, research indicates that accurate knowledge about the COVID-19 virus significantly correlates with positive vaccine attitudes among HCWs, emphasising the crucial role of education in shaping vaccination behaviour.^41^ A study by Marcu et al. underscored that proper knowledge increases HCWs’ willingness to receive vaccines and recommend them to others.^42^

Vaccine acceptance rates among low- and middle-income countries (LMICs) appear to be less influenced by factors such as vaccine access, costs and vaccine awareness.^43^ This is consistent with the findings of our study, as neither vaccine accessibility nor affordability emerged as reasons for not receiving vaccination among participants. Notably, in South Africa, the vaccine was readily accessible in both public and private healthcare facilities at no cost to individuals; such factors contributed to the equitable accessibility of the COVID-19 vaccine. Additionally, health workers were prioritised in the vaccine dispensation programme; thus, most medical laboratory workers had easy access to the vaccine. Our findings support the conceptual framework on drivers of vaccine hesitancy.^14^

Health workers hold substantial influence in encouraging their families and communities to pursue vaccination. Therefore, it is crucial to examine their vaccine attitudes and promptly address any concerns they may harbour as HCWs who show confidence in the safety and efficacy of the COVID-19 vaccine are more inclined to advocate for its adoption among both colleagues and patients.

## Conclusion

The prevalence of COVID-19 vaccine acceptance among NHLS workers is notably high. Our study revealed that older NHLS medical laboratory workers are more likely to receive the COVID-19 vaccine compared to their younger counterparts. Notably, while vaccine accessibility and affordability were not barriers to COVID-19 vaccination, vaccine hesitancy among non-vaccinated participants stemmed from a lack of trust in the vaccine research, safety, potential long-term effects and uncertainties surrounding side effects.

### Recommendations

We recommend a proactive approach of raising awareness on vaccination regularly to improve vaccine acceptance and uptake of available vaccination services, including basic information on the importance of vaccination as part of continuous learning for medical laboratory workers. We recommend educating medical laboratory workers on various aspects of vaccination, including COVID-19 vaccine research, safety, potential long-term effects and uncertainties surrounding side effects, to address vaccine hesitancy among those medical laboratory workers who remain hesitant to receive necessary vaccines. We recommend further follow-up in-depth research on vaccine acceptance and hesitancy among medical laboratory workers in South Africa.

### Limitations

Our study had some limitations. Initially, it was conducted as a cross-sectional study, preventing the establishment of causal relationships between COVID-19 vaccine acceptance and independent variables. Additionally, the study had a low response rate, thereby limiting the generalisability of its findings.
